# Risk factors for hidden blood loss in unilateral biportal endoscopic lumbar spine surgery

**DOI:** 10.3389/fsurg.2022.966197

**Published:** 2022-08-15

**Authors:** Sijia Guo, Haining Tan, Hai Meng, Xiang Li, Nan Su, Linjia Yu, Jisheng Lin, Ning An, Yong Yang, Qi Fei

**Affiliations:** Department of Orthopedics, Beijing Friendship Hospital, Capital Medical University, Beijing, China

**Keywords:** unilateral biportal endoscopy (UBE), hidden blood loss (HBL), minimally invasive spine surgery, lumbar disorder, risk factors

## Abstract

**Background:**

Unilateral biportal endoscopic (UBE) spine surgery is a minimally invasive procedure for treating lumbar disorders. Hidden blood loss (HBL) is easily ignored by surgeons because blood loss is less visible. However, there are limited studies on HBL in UBE spine surgery. This study aimed to evaluate HBL and its possible risk factors in patients undergoing UBE spine surgery.

**Methods:**

Patients with lumbar disc herniation or lumbar spinal stenosis who underwent unilateral biportal endoscopic surgery between December 2020 and February 2022 at our hospital were retrospectively analyzed. Patient demographics, blood loss-related parameters, and surgical and radiological information were also collected. Pearson or Spearman correlation analysis was conducted to determine the association between clinical characteristics and HBL. Multivariate linear regression analysis was used to determine the independent risk factors for HBL.

**Results:**

Fifty-two patients (17 males and 35 females) were retrospectively enrolled in this study. The mean total blood loss (TBL) volume was 434 ± 212 ml, and the mean HBL volume was 361 ± 217 ml, accounting for 77.9% of the TBL in patients who underwent UBE surgery. Multivariate linear regression analysis revealed that HBL was positively associated with operation time (*P* = 0.040) and paraspinal muscle thickness at the target level (*P* = 0.033).

**Conclusions:**

The amount of HBL in patients undergoing UBE surgery should not be neglected. Operation time and paraspinal muscle thickness at the target level may be independent risk factors for HBL.

## Introduction

Unilateral biportal endoscopy (UBE) is an emerging minimally invasive surgical procedure for the treatment of lumbar disorders. Spine surgery is favored by spine surgeons because of the lower rate of surgical injury, quicker postoperative recovery, and limited influence on spinal stability (1). The efficacy and safety of UBE have been confirmed in previous studies (2–5). However, the amount of blood loss is easily underestimated by spine surgeons because of continuous irrigation and the blood infiltrating into the soft tissue or remaining in the dead space of the surgical channel.

HBL was first proposed by Sehat et al. (6) and has attracted increasing attention from surgeons. HBL is common in minimally invasive spine surgeries. Jiang et al. (7) compared the clinical outcomes between UBE and percutaneous endoscopic lumbar discectomy (PELD) in the treatment of patients with lumbar disk herniation and found that the HBL volume in PELD and UBE were 30.64 ± 22.29 ml and 195.62 ± 130.44 ml, respectively. Wang et al. (8) evaluated the mean HBL volume in patients undergoing UBE surgery for lumbar degenerative diseases to be 469.5 ± 195.3 ml. Moreover, accurate evaluation of hidden blood loss (HBL) during UBE surgery is helpful for reducing perioperative complications and ensuring patient safety. However, to our knowledge, there is limited literature on HBL and its risk factors in UBE surgery for lumbar disorders. Therefore, this study aimed to estimate the amount of HBL and its risk factors in patients with lumbar disorders who underwent UBE surgery.

## Patients and methods

This retrospective study was approved by the Ethics Committee of Beijing Friendship Hospital, Capital Medical University. Informed consent was obtained from all participants. Fifty-two patients diagnosed with lumbar spinal stenosis or lumbar disc herniation were included in this study from December 2020 to February 2022. The exclusion criteria were as follows: (1) age <18 years old; (2) presence of lumbar spine tumor, infection, or trauma; (3) use of anticoagulant or antiplatelet drugs; (4) presence of liver or kidney dysfunction, abnormal bleeding, or abnormal coagulation function; (5) presence of scoliosis, ankylosing spondylitis, or other spinal deformities; and (6) incomplete medical records.

### Data collection

Clinical data, including sex, age, height, weight, body mass index (BMI), hypertension, diabetes, coronary heart disease (CHD), history of smoking, history of alcohol use, American Society of Anesthesiologists (ASA) classification, operation time, surgical level, and disc dissection were systematically collected.

Triglyceride (TG), serum total cholesterol (TC), low-density lipoprotein (LDL), high-density lipoprotein (HDL), hemoglobin (Hb), hematocrit (Hct), platelet (PLT), albumin (ALB), prothrombin time (PT), activated partial thromboplastin time (APTT), international normalized ratio (INR), D-dimer, and fibrinogen (Fbg) levels were recorded before surgery. Hct, ALB, Hb, PLT, and drainage levels were recorded on postoperative day 1.

**Figure 1:**
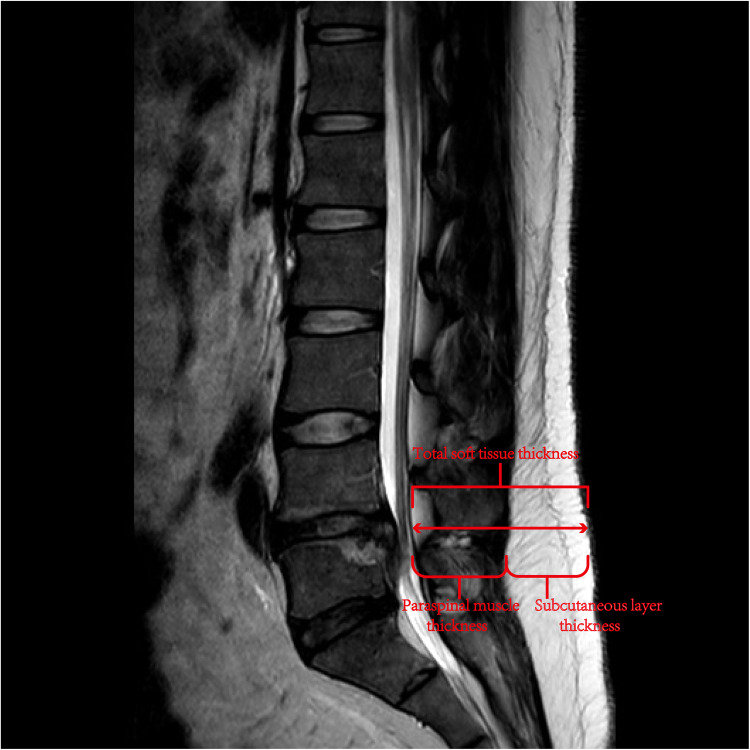
Diagram illustrating the method used to measure the thickness of total soft tissue, paraspinal muscle and subcutaneous layer at the level of L5 through sagittal view on T2-weighted MRI.

The total soft-tissue thickness, subcutaneous layer thickness, and paraspinal muscle thickness at the target level were independently measured by two experienced radiologists using lumbar MRI images (Figure 1). The MRI measurements have demonstrated good internal consistencies with Cronbach's alpha ranging from 0.86 to 0.90.

### Calculation of blood loss

Patients' blood volume (PBV) was calculated using the Nadler formula (9): *k*_1_ = 0.3669, *k*_2_ = 0.03219, and *k*_3_ = 0.6041 for males and *k*_1_ = 0.3561, *k*_2_ = 0.03308, and *k*_3_ = 0.1833 for females.


$$\mathrm{PBV}=k_{1}\times \mathrm{Heigh}t^{3}\left(m\right)+k_{2}\times \mathrm{Weight}\left(\mathrm{kg}\right)+k_{3}$$

Total blood loss (TBL) was calculated using Gross formula (10):

$$\mathrm{TBL}=\frac{\mathrm{PBV}\times \left(\mathrm{Hc}t_{\;\mathrm{post}}-\mathrm{Hc}t_{\;\mathrm{pre}}\right)}{\mathrm{Hc}t_{\mathrm{ave}}}$$

Hct_pre_ is the Hct on preoperative day 1, Hct_post_ is the Hct on postoperative day 1 and Hct_ave_ is the average of Hct_pre_ and Hct_post_.

Thus, the HBL was calculated as follows:


Visiblebloodloss(VBL)=intraoperativebloodloss+postoperativedrainage

HBL=TBL−VBL

### Statistical analysis

Categorical variables were grouped and presented as numerical values, and continuous data were presented as mean ± standard deviation. Pearson's correlation analysis, Spearman's correlation analysis, and multiple linear regression were used to determine the factors associated with HBL, including continuous and categorical variables respectively. Statistical significance was set at *P* < 0.05. All data analyses were performed using SPSS v25.0 software (IBM Corp., Armonk, NY, United States).

## Results

Fifty-two consecutive patients (17 males and 35 females) were retrospectively enrolled in this study. The demographic characteristics of the participants are summarized in Table 1. The mean age was 61.2 ± 14.3 (range, 26–84) years, and the mean BMI was 25.8 ± 4.3 kg/m^2^. Regarding lumbar disorders, 27 patients had lumbar disk herniation and 35 had lumbar spinal stenosis. With respect to comorbidities, 27, 11, and 6 patients had hypertension, diabetes, and CHD, respectively. The mean surgery time was 132.2 ± 46.0 min. In total, 56 levels were operated, of which 2 were at L2–3, 6 at L3–4, 29 at L4–5, and 19 at L5–S1. Forty-eight patients underwent UBE surgery at a single level, and four patients underwent surgery at double levels. In terms of ASA classification, 2, 38, and 12 patients had a physical status classification of I, II, and III, respectively. The mean total soft tissue thickness, paraspinal muscle thickness, and subcutaneous layer thickness measured using MRI were 5.5 ± 1.1, 3.6 ± 0.6, 1.8 ± 1.0 cm, respectively. The mean PBV was 4.1 ± 0.7 L, mean TBL volume was 434.0 ± 212.0 ml, mean VBL volume was 72.5 ± 41.0 ml, mean HBL volume was 361.4 ± 216.8 ml (77.9% of the TBL). The mean amounts of Hct and Hb lost were 4.2 ± 2.0 and 11.9 ± 7.2 g/L, respectively. Postoperative Hb and Hct levels were significantly lower than the preoperative levels (*P* < 0.001 for both). Meanwhile, eight patients developed anemia (seven mild and one moderate) after UBE surgery, accounting for 15.4% of all the patients. None of the patients received perioperative transfusions. No significant difference was found in HBL between the lumbar disc herniation and lumbar spinal stenosis groups.

**Table 1:** Patients’ demographics and clinical information.

Parameters	Statistics
Total patients (*n*)	52
Sex (*n*)	
Female	35 (67.3%)
Age, year	61.2 ± 14.3
BMI, kg/m^2^	25.8 ± 4.3
Height, cm	162.8 ± 8.4
Weight, kg	68.5 ± 13.4
Hypertension (*n*)	27 (51.9%)
Diabetes mellitus (*n*)	11 (21.2%)
CHD (*n*)	6 (11.5%)
Smoking (*n*)	6 (11.5%)
Drinking (*n*)	2 (3.8%)
Diseases groups	
Lumbar disc herniation	27 (51.9%)
Lumbar spinal stenosis	25 (48.1%)
Operation level	
L2–L3	2(3.6%)
L3–L4	6(10.7%)
L4–L5	29(51.8%)
L5–S1	19(33.9%)
Single-level operation (*n*)	4 (7.7%)
Double-level operation (*n*)	48 (92.3%)
Tranexamic acid (*n*)	47 (90.4%)
Lumbar disk dissection (*n*)	25 (48.1%)
ASA classification (*n*)	
I	2(3.8%)
II	38(73.1%)
III	12(23.1%)
IV	0
Surgery time, min	132.2 ± 46.0
PBV, L	4.1 ± 0.7
TBL, ml	434.0 ± 212.0
VBL, ml	72.5 ± 41.0
HBL, ml	361.4 ± 216.8
Preoperative Hb, g/L	136.0 ± 15.1
Postoperative Hb, g/L	124.0 ± 15.0
Hb loss, g/L	11.9 ± 7.2
Preoperative Hct	41.2 ± 4.3
Postoperative Hct	37.0 ± 4.2
Hct loss	4.2 ± 2.0
Preoperative ALB, g/L	38.7 ± 3.2
Postoperative ALB, g/L	34.9 ± 3.1
Alb loss, g/L	3.8 ± 2.6
Preoperative Platelet, g/L	241.6 ± 72.1
Preoperative PT, s	11.6 ± 1.1
Preoperative APTT, s	27.4 ± 3.1
Preoperative Fibrinogen, g/L	2.6 ± 0.6
Preoperative D-dimer, μg/ml	0.4 ± 0.5
Preoperative TC	4.7 ± 1.2
Preoperative TG	1.8 ± 1.7
Preoperative LDL	2.7 ± 0.7
Preoperative HDL	1.1 ± 0.3
Soft tissue thickness, cm	5.5 ± 1.1
Paraspinal muscle thickness, cm	3.6 ± 0.6
Subcutaneous layer thickness, cm	1.8 ± 1.0

BMI, body mass index; CHD, coronary heart disease; ASA, American society of anesthesiologists; PBV, patients’ blood volume; TBL, total blood loss; VBL, visible blood loss; HBL, hidden blood loss; Hb, hemoglobin; Hct, hematocrit; Alb, albumin; PT, prothrombin time; APTT, activated partial thromboplastin time; INR, international normalized ratio; TC, total cholesterol; TG, triglyceride; LDL, low-density lipoprotein; HDL, high-density lipoprotein.

The Pearson and Spearman correlation analyses results are shown in Table 2. The analyses showed that the paraspinal muscle thickness at the target level was related to HBL (*P* < 0.05). The following factors with *P* < 0.10 were included in the multivariate linear regression analysis to identify the independent risk factors for HBL: operation time (*P* = 0.072), paraspinal muscle thickness (*P* = 0.025), preoperative Hct level (*P* = 0.055), preoperative Fbg level (*P* = 0.074), and preoperative Hb level (*P* = 0.084), and the results showed that paraspinal muscle thickness (*P* = 0.033) and operation time (*P* = 0.040) were significant independent risk factors (Table 3).

**Table 2:** Correlation analysis between clinical factors and HBL.

Parameters	*P*	Correlation
Sex	0.435	−0.111
Age	0.638	0.067
BMI	0.736	−0.048
Height	0.825	−0.031
Weight	0.594	−0.076
Hypertension	0.241	−0.165
Diabetes mellitus	0.178	−0.190
CHD	0.672	0.060
Smoking	0.693	−0.056
Drinking	0.743	−0.047
Diseases groups	0.836	0.029
Operation level	0.803	0.037
single/double levels	0.308	0.144
Tranexamic acid	0.530	0.089
Lumbar disk dissection	0.607	0.073
ASA classification	0.139	−0.208
Surgery time	0.072	0.251
Preoperative Hb	0.084	0.220
Preoperative Hct	0.055	0.268
Preoperative ALB	0.271	0.155
Preoperative Platelet	0.543	−0.086
Preoperative PT	0.592	0.078
Preoperative APTT	0.218	−0.177
Fibrinogen	0.074	−0.255
D-dimer	0.227	−0.174
Preoperative TC	0.655	0.063
Preoperative TG	0.114	0.222
Preoperative LDL	0.713	0.052
Preoperative HDL	0.719	−0.051
Soft tissue thickness	0.274	0.155
Subcutaneous layer thickness	0.897	−0.018
**Paraspinal muscle thickness**	**0.025**	**0.310**
Paraspinal muscle ratio	0.593	0.076

Value in bold indicates statistical significance.

BMI, body mass index; CHD, coronary heart disease; ASA, American society of anesthesiologists; PBV, patients’ blood volume; TBL, total blood loss; VBL, visible blood loss; HBL, hidden blood loss; Hb, hemoglobin; Hct, hematocrit; Alb, albumin; PT, prothrombin time; APTT, activated partial thromboplastin time; INR, international normalized ratio; TC, total cholesterol; TG, triglyceride; LDL, low-density lipoprotein; HDL, high-density lipoprotein.

**Table 3:** Multivariate linear regression analysis on risk factors of HBL.

Coefficients^a^	Unstandardized *β*	SE	Standardized *β*	*t*	*P*
Constant	−198.707	198.24		−1.002	0.321
Paraspinal muscle thickness	107.052	48.662	0.294	2.2	0.033
Operation time	1.278	0.606	0.282	2.109	0.040

^a^ Dependent variable: hidden blood loss (ml).

## Discussion

Recently, UBE surgery has shown advantages in the treatment of lumbar disorders due to the less trauma, quick postoperative recovery, and less influence on spinal stability. Although previous studies have elaborated on the complications following UBE surgery, spine surgeons have underestimated HBL in UBE surgery. Wang et al. (8) retrospectively analyzed patients who underwent UBE surgery and reported an HBL volume of 469.5 ± 195.3 ml, accounting for 57.6% of TBL. Age, number of fusion levels, ASA classification, surgery time, PBV, TBL, postoperative Hct, Hct loss, and fibrinogen level were independent risk factors for HBL. Our findings showed a mean HBL of 361.4 ± 216.8 ml, accounting for 77.9% of the TBL in patients who underwent UBE for lumbar disorders. Similar with previous studies on HBL in spine surgery (7, 8), the amount of HBL during surgery was significantly higher than that of VBL. Excessive HBL not only increases the incidence of perioperative complications but also prolongs patient recovery time. The purpose of this study aimed to explore the risk factors of HBL in UBE spine surgery. And we hope that our finding could help spine surgeons identify potential groups of patients at high risk of bleeding and pay more attention to intraoperative hemostasis and perioperative blood loss management during minimally invasive surgery, thereby reducing perioperative complications and ensuring patient safety.

Although some theories have been proposed to explain HBL, the mechanism underlying HBL has not yet been clarified. Bivariate correlation and multiple linear regression analyses were performed to determine the risk factors for HBL. Our results showed that paraspinal muscle thickness at the target level and operation time were independent risk factors for HBL. We found that the thicker the paraspinal muscle at the target level, the larger the amount of HBL. There are two possible explanations for this observation. First, muscle tissue is rich in blood supply; paraspinal muscle thickness at the target level indicated the need for longer working channels to be established during UBE surgery, increasing the wound and intraoperative bleeding. Second, paraspinal muscle tissue thickness might be related to large blood infiltration, allowing more blood to penetrate the tissue space. This finding is consistent with those of previous studies on HBL in patients undergoing oblique lateral interbody fusion surgery or cervical open-door laminoplasty (11, 12). It might be important to evaluate the thickness of the paraspinal muscle at the target level of the patient using MRI before surgery. Surgeons should pay attention to the risk of excessive HBL, especially in patients with thick paraspinal muscle tissue, and achieve satisfactory hemostasis of muscle tissue as much as possible. However, the thickness of the subcutaneous layer, total soft tissue, and proportion of paraspinal muscle in the soft tissue did not show any significant relationship with HBL in this study. This may be related to the small sample size of the study. Further research is required to clarify the effects of tissue type on HBL.

Our study demonstrated that operative time was an independent risk factor for HBL. This finding is consistent with the results of previous studies (8, 13). During the UBE surgery, saline was used to irrigate and achieve good surgical vision. Continuous irrigation with a large amount of fluid flushes out the seeping blood through the soft tissue and bone surfaces. With the extension of the operation time, the blood flushed increased. Therefore, surgeons might need to be alert to the potential for excessive HBL during UBE surgery, especially if the operation time is too long. Meanwhile, a certain pressure or rapid flow of saline during irrigation might help reduce blood loss during surgery (14, 15).

The current study has some limitations. First, it was a retrospective study with a relatively small sample size and a lack of control group. Future prospective studies with larger sample sizes are required to confirm these results. Second, our study did not enroll patients undergoing fusion surgery, and the amount of TBL and related risk factors might differ from those in previous studies. Further research is required to explore the impact of spinal fusion on HBL during UBE surgery. Besides, considering that postoperative drainage might be affected by intraoperative irrigation, the calculation of VBL and HBL might be slightly biased.

## Conclusion

This study showed that a large amount of HBL occurred during the UBE procedure for treating lumbar disc herniation or spinal stenosis. Operation time and paraspinal muscle thickness at the target level were independent risk factors for HBL in patients with lumbar disorders who underwent UBE surgery.

## Data Availability

The raw data supporting the conclusions of this article will be made available by the authors, without undue reservation.

## References

[1] Pao JL Lin SM Chen WC Chang CH. Unilateral biportal endoscopic decompression for degenerative lumbar canal stenosis. J Spine Surg. (2020) 6:438–46. 10.21037/jss.2020.03.08 32656381 PMC7340817

[2] Heo DH Sharma S Park CK. Endoscopic treatment of extraforaminal entrapment of L5 nerve root (far out syndrome) by unilateral biportal endoscopic approach: technical report and preliminary clinical results. Neurospine. (2019) 16:130–7. 10.14245/ns.1938026.013 30943715 PMC6449829

[3] Eum JH Heo DH Son SK Park CK. Percutaneous biportal endoscopic decompression for lumbar spinal stenosis: A technical note and preliminary clinical results. J Neurosurg Spine. (2016) 24:602–7. 10.3171/2015.7.SPINE15304 26722954

[4] Akbary K Kim JS Park CW Jun SG Hwang JH. Biportal endoscopic decompression of exiting and traversing nerve roots through a single interlaminar window using a contralateral approach: Technical feasibilities and morphometric changes of the lumbar canal and foramen. World Neurosurg. (2018) 117:153–61. 10.1016/j.wneu.2018.05.111 29857220

[5] Choi DJ Jung JT Lee SJ Kim YS Jang HJ Yoo B. Biportal endoscopic spinal surgery for recurrent lumbar disc herniations. Clin Orthop Surg. (2016) 8:325–9. 10.4055/cios.2016.8.3.325 27583117 PMC4987318

[6] Sehat KR Evans R Newman JH. How much blood is really lost in total knee arthroplasty? – Correct blood loss management should take hidden loss into account. Knee. (2000) 7:151–5. 10.1016/S0968-0160(00)00047-8 10927208

[7] Jiang HW Chen CD Zhan BS Wang YL Tang P Jiang XS. Unilateral biportal endoscopic discectomy versus percutaneous endoscopic lumbar discectomy in the treatment of lumbar disc herniation: A retrospective study. J Orthop Surg Res. (2022) 17:30. 10.1186/s13018-022-02929-5 35033143 PMC8760683

[8] Wang H Wang K Lv B Li W Fan T Zhao J Analysis of risk factors for perioperative hidden blood loss in unilateral biportal endoscopic spine surgery: A retrospective multicenter study. J Orthop Surg Res. (2021) 16:559. 10.1186/s13018-021-02698-7 34526051 PMC8442349

[9] Nadler SB Hidalgo JH Bloch T. Prediction of blood volume in normal human adults. Surgery. (1962) 51:224–32. 21936146

[10] Gross JB. Estimating allowable blood loss: Corrected for dilution. Anesthesiology. (1983) 58:277–80. 10.1097/00000542-198303000-00016 6829965

[11] Zhu L Zhang L Shan Y Feng X Zhang W. Analysis of hidden blood loss and its risk factors in oblique lateral interbody fusion surgery. Clin Spine Surg. (2021) 34:E501–E5. 10.1097/BSD.0000000000001177 33783370

[12] Jiang C Chen TH Chen ZX Sun ZM Zhang H Wu YS. Hidden blood loss and its possible risk factors in cervical open-door laminoplasty. J Int Med Res. (2019) 47:3656–62. 10.1177/0300060519856987 31234677 PMC6726792

[13] Zhang R Xing F Yang Z Lin G Chu J. Analysis of risk factors for perioperative hidden blood loss in patients undergoing transforaminal lumbar interbody fusion. J Int Med Res. (2020) 48:300060520937848. 10.1177/0300060520937848 32772761 PMC7418255

[14] Zhang H Zhou C Wang C Zhu K Tu Q Kong M Percutaneous endoscopic transforaminal lumbar interbody fusion: technique note and comparison of early outcomes with minimally invasive transforaminal lumbar interbody fusion for lumbar spondylolisthesis. Int J Gen Med. (2021) 14:549–58. 10.2147/IJGM.S298591 33654422 PMC7910530

[15] Hu A Gu X Guan X Fan G He S. Epidural versus intravenous steroids application following percutaneous endoscopic lumbar discectomy. Medicine. (2018) 97:e0654. 10.1097/MD.0000000000010654 29718884 PMC6392748

